# Three Types of Intimate Relationships among Individuals with Chronic Pain and a History of Trauma Exposure

**DOI:** 10.3390/healthcare5040068

**Published:** 2017-09-29

**Authors:** Carissa van den Berk-Clark, Terri L. Weaver, F. David Schneider

**Affiliations:** 1 Department of Family and Community Medicine, Saint Louis University School of Medicine, St. Louis, MO 63104, USA; 2 Department of Psychology, Saint Louis University School of Arts and Sciences, St. Louis, MO 63110, USA; weavert@slu.edu; 3 Department of Family and Community Medicine, University of Texas Southwestern Medical Center, Dallas, TX 75390, USA; David.Schneider@utsouthwestern.edu

**Keywords:** chronic pain, social support, PTSD, trauma

## Abstract

Individuals with chronic pain often have psychiatric disorders, such as depression and posttraumatic stress disorder (PTSD), which can affect their intimate relationship satisfaction and stability. Little is known about the nature of support stemming from chronic pain patients’ intimate relationships, and therefore, this study sought to: (1) use cluster modeling to construct specific intimate relationship groups based on types of support patients receive, and (2) determine if there is a relationship between support type and PTSD, chronic pain, anxiety, and depression. Ward’s method of cluster analysis in Stata was used to create groups based on the level of informational, affirmation, confident, emotional, and fun support received from chronic pain patients’ most intimate relationship. Three types of support were identified: high (type 1, n = 17), high emotional/low instrumental (type 2, n = 9), and unstable (type 3, n = 15). Types 1 and 3 included more family members (Type 1: 100%, Type 2: 93%), than type 2 (77%). Type 2 patients experienced more trauma (Mean = 9.4 ± 1.7 vs. 7.5 ± 0.88 for types 1 and 3) and were significantly more likely to have PTSD (X^2^ = 7.91, *p* < 0.05. Patients with low familial support may also benefit from PTSD screening and referral but further study is needed.

## 1. Introduction

Chronic pain is one of the most common symptoms treated by primary care physicians (PCPs) in the United States. With an estimated 126.1 million adults suffering from some type of chronic pain in the past three months [1], these patients are more likely to have chronic health conditions, less education, less likely to work due to disability, are more likely to report daily feelings of anxiety (45.3%), depression (56.8%), and fatigue (63.7%) [2], and are often overweight and engage in substance misuse [3]. A number of studies have shown that the individuals with chronic pain have dealt with significant exposure to traumatic events in their lifetimes, which has led to a much higher likelihood of posttraumatic stress disorder (PTSD) [4]. All of these factors have been shown to affect how individuals with chronic pain relate to individuals in their social support networks, especially those with which they have the closest relationship(s).

Meanwhile, large multi-site studies, like the Adverse Childhood Experiences study, have shown how childhood exposure to traumatic events is associated with a marked and graded increased risk of future health problems. Kaiser Permanente healthcare member patients exposed to four or more childhood traumas engaged in riskier health behaviors (e.g., alcohol and/or drug abuse, suicide attempts, smoking, multiple sexual partners, physical inactivity, and overeating) and had more negative health and mental health outcomes (e.g., ischemic heart disease, cancer, chronic lung disease, skeletal fractures, liver disease, depression, anxiety, eating disorders, and PTSD) [5]. Further, studies have also shown significant evidence of a link between potentially traumatic events (including events which is not occurring in childhood), risky health behaviors and health problems (for review, see [6]), particularly coronary artery disease [7], lung function indicating airway obstruction [8], and chronic pain [4] in military personnel, veterans, and civilian populations [9]. Although a number of studies have shown an independent association between traumatic event exposure and health (for review, see [9]), many other studies have shown that PTSD is the mediating pathway.

Given that trauma-exposed chronic pain patients often have limited functioning due to their pain and a higher burden of chronic disease, they may need to rely more heavily on their social support networks (for day-to-day instrumental and emotional support) and clinicians (for health services). For clinicians, the quality of patients’ social support networks are of significant concern; these network members encourage or discourage healthy or unhealthy behaviors and may support compliance with, and utilization of, healthcare. The inability to relate to an intimate partner may also more broadly define the patient’s relationships in general, including the patient-healthcare provider relationship. That is, patients with PTSD who are unable to trust, have high levels of irritability, and anger easily, have difficulties with impulse control [10], and may make create interpersonal challenges to the provision of quality care.

To better understand both chronic pain patients and their social support networks, we conducted detailed, semi-structured interviews with 41 chronic pain patients. In doing so, we sought to answer the following research questions:

(1) What types of social support and social stressors exist for trauma-exposed chronic pain patients in their most intimate relationships?
(2) Do some trauma-exposed chronic pain patients experience more social support and stressors than other trauma-exposed patients within their relationships?
(3) Is the quality of these relationships significantly related to other outcomes of traumatized chronic pain patients, including level of pain, anxiety, PTSD and depression?

## 2. Materials and Methods

### 2.1. Participants

Forty-one patients were recruited from four primary care clinics affiliated with a large regional hospital system in St. Louis, Missouri to participate in detailed, semi-structured interviews. Together, the four sites service patients and families from diverse socioeconomic, ethnic/racial, and urban/rural backgrounds. Inclusion criteria for the sample included being over the age of 18, a current patient at one of the four clinics, and a diagnosis of chronic pain (lasting three months or longer). Exclusion criteria included individuals who cannot speak English and who lacked the cognitive capacity to provide consent, who did not have a chronic pain condition, and who had not been exposed to a DSM 5 qualifying traumatic event. Cognitive capacity was determined by a review of the electronic medical record, and if the interviewer detected possible cognitive defects, the Mini-Mental Status Exam (cutoff = 27) was administered [11].

### 2.2. Data Collection

After participants agreed to participate in the study, a trained research assistant interviewed participants about their close social networks. Study participants were then asked to complete a self-administered survey that assesses sociodemographic, health, and mental health characteristics. All of the interviews were conducted by master’s level professional research assistants with a background in either social work or public health. Research assistants read the survey questions to participants with reading difficulties and recorded their responses. This study was approved by the internal review board at Saint Louis University School of Medicine (IRB Number 22321).

### 2.3. Measures

#### 2.3.1. Social Networks

Adapted from the Social Convoy Model [12,13], research participants were shown three embedded circles, which represent their inner, middle, and outer social relationships. They were then instructed to write the initials of people in the different circles based on how close they feel to that person, with the inner circle being their closest relationships. Two different social network instruments were utilized to assess stressfulness of network relationships, network function (i.e., capacity of network members to fulfill subjects’ needs), and the role of the relationship in encouraging healthy behavior: the Social Network Assessment Instrument [14] and Barrera’s Arizona Social Support schedule [15]. Since individuals can have different kinds and levels of stress in their social networks, an open-ended question was used to clarify how the network member either enhanced or reduced stressors. This study focused on the social network member that participants identified as their most intimate relationship.

#### 2.3.2. Health and Mental Health Characteristics 

Health and mental health characteristics included both physical (level of pain) and mental health (depression, anxiety, and PTSD). Level of pain was assessed using a 1–10 scale with 10 being the worst pain ever experienced and 1 being no pain. We utilized the Patient Health Questionnaire (PHQ-9) to assess symptoms of depression and the Beck Anxiety Inventory—Primary Care (BAI-PC) to assess symptoms of anxiety. The psychometric properties of both instruments are well established (PHQ-9 alpha = 0.86–0.89, test-retest reliability (r = 0.84), cutoff ≥ 15) [16], (BAI-PC alpha = 0.90, cutoff ≥ 5) [17]. PTSD and trauma were assessed using the Posttraumatic Diagnostic Scale and Trauma Screen [18]. This instrument was selected because it: (1) focuses on two symptom categories known to effect interpersonal relationships, (namely, avoidance and hyperarousal), (2) has demonstrated utility among primary care populations [19], and (3) has an extensive list of applicable traumatic events. We used a cut-off score of >3 for PTSD diagnosis [20].

### 2.4. Analysis

Complete patient PCP and chart data was extracted to Stata v. 13.0 (Stata Inc.) [21] and demographics, trauma exposure, mental health status, and the level of pain were summarized or analyzed. To establish the types of social support networks, we utilized Ward’s Method of cluster analysis in Stata on standardized variables for network function including instrumental support (i.e., economic support or specific resources such as access to a car), informational support, affirmation support, confident support, emotional support, and fun support. Ward’s method was used because it equalizes subgroup size, and makes it possible to produce three different spherical subgroups of participants, which is helpful when the sample size is small [22]. To detect differences between clusters, Chi Square, *t*-tests and One­Way analysis of variance (ANOVA) were used.

## 3. Results

### 3.1. Demographic and Descriptive Characteristics

Participants were mostly female (61.0%) and had a mean age of 52.9 (SD = 12.7). About half of the participants were White (51.2%) and the other half African American (48.8%; Table 1). Thirty percent of the group was either married or cohabiting with their intimate partner. For the most part, participants were not employed (either full time or part time) and only 17.1% had a household income at $50,000 or higher.

One hundred percent of participants had experienced a potentially traumatic event with the number of events ranging from 1 to 19. Reported traumatic events included combat (2.4%), assault by stranger (46%), assault by someone known (63%), sexual assault by a stranger (20%), sexual assault by known someone (29%), life threatening accident (56%), natural disasters (27%), life threatening illness (44%), torture (12%), kidnapping (20%), and incest (27%). the mean number of traumatic events was 7.9 (SD = 3.9, range 1–19). About 27% of the sample had a probable diagnosis of PTSD, 24% had depression and 59% had anxiety. Mean pain score was 5.1 (SD = 2.1).

### 3.2. Subtypes of Social Support

Analysis identified three clusters of social support. These types included Type 1: High support overall, Type 2 high emotional/instrumental support and Type 3 unstable support. Type 1, in which 100% of its members experienced instrumental, informational, affirmation, confident, emotional, and fun support from their most intimate relationships (see Table 2). Type 1 intimate relationships were all biological/familial and included the following: 41% spouse, 29% parent, 18% biological child and 12% sibling. Compared to types 2 and 3, type 1 relationships were much less stressful (29% said not at all, 47% said a little bit, and 24% said somewhat). Type 1 participants trusted their most intimate relationship either quite a bit (12%) or extremely (88%). Only 17% of type 1 intimate relationships were somewhat, quite a bit or extremely critical, and there were fewer demands from the relationship and no verbal or physical aggression.

Type 2 had the most non-familial individuals as their most intimate relationship (33%). These relationships had high levels of informational (100%) and affirmation support (100%), and most of them provided confidant (78%) and emotional support (89%). Nearly one quarter (22%) of the most intimate relationships in type 2 were considered quite stressful or extremely stressful. These relationships were less trusting than type 1 with 22% trusting their most intimate relationship not at all or a little bit. Nevertheless, type 2 participants did not find the person who was in the intimate relationship with them to be critical or disapproving of them, did not experience too many demands from this person nor this person to be verbally aggressive (only 22% are somewhat verbally aggressive), but some found it challenging (22%) to measure up to the expectations of their most intimate relationship.

Type 3 had social support networks more biological/familial in nature than type 2 with only 7% stating that their most intimate relationship was with a friend, roommate, or other person. Type 3 individuals received unstable support across the board with only 45% having instrumental support (slightly higher than type 2), 13% having informational support, 27% affirmation support, 47% confidant support, 53% emotional support, and 40% fun support. Type 3 was the only group to experience verbal aggression from their most intimate relationship (20% somewhat, 13% quite a bit, and 7% extremely). This higher tendency toward verbal aggression was also reflected within other relationship stressors: 53% of this group thought their most intimate relationship made their life quite a bit more or extremely stressful; 40% did not trust their most intimate relationship; 74% thought that their most intimate relationship was too critical or disapproving of them; 53% thought that they did not measure up; and, 40% thought that this relationship was too demanding. Open-ended responses in Table 3 also reflected a significantly higher amount of relationship breakdown in this group with intimate partners more likely to resent, criticize, threaten, exploit, misunderstand, and lie to each other.

### 3.3. Demographic Differences in the 3 Subtypes

Demographic factors were explored across each of the three social support subtypes. Type 3 was older than the other groups (mean age 57.0 (SD = 3.4) vs. mean age 47.7 (SD = 4.5) type 2, mean age 51.8 (SD = 2.8) type 1). More males were in types 2 and 3. Type 1 members were more likely to be married than other groups (52.9% vs. 33.3% types 2 and 3) and to be employed (35% vs. 11.1% types 2 and 3), while type 3 members tended to have a higher income. Fewer African Americans had type 1 subtype relationships than Whites.

### 3.4. Mental Health and Pain Symptom Differences in 3 Subtypes

Type 2 reported experiencing more potentially traumatic events (mean 9.4 (SD = 1.7) vs. mean 7.5 (SD = 0.9) for types 1 and 3 and was significantly more likely to have PTSD (55.6% vs. 33.3% type 3 and 5.9% type 1) (X^2^ = 7.91, *p* < 0.05. The level of pain among subtypes was similar with 5.2 (SD = 0.6) type 1, 5.4 (SD = 0.8) type 2 and 4.8 (SD = 0.5) type 3. Although non-significant, individuals with type 2 intimate relationships were more likely to have anxiety (77.8% vs. 52.8% for types 1 and 3) and depression (44.4% vs. 18.8% for types 1 and 3).

### 3.5. Qualitative Differences between the 3 Subtypes

Three quotes are displayed in Table 4, which correspond to interview focuses on stress, verbal aggression, positive influence and contribution to healthy choices. The table shows that type 1 relationships tend to involve minor stressors, like small differences in opinion or concern about intimate supports safety during routine daily events (i.e., driving). Those with type 1 relationships tend to get significant pressure to make positive health choices across a variety of different domains, not just when it comes to taking medication or following up with care. When unhealthy choices are made, it was usually as a temporary indulgence. Type 2 relationships tended to be more stressful, but the stress was more related to worry over the intimate relations health. More arguments were reported among type 2 than type 1 and those arguments were less understanding of mutual similarities and responsibilities. Positive influence among type 2 focused more on provision of emotional support and reminders to take medication or followup with care. Type 3 relationship stress was much more toxic, and seemed to include resentment on the part of intimate relations about individual functioning. Verbal aggression highlighted significant lack of trust in relationships and lack of reciprocation. However, intimate supports did play a significant role in keeping the person on track with medications and followup care.

## 4. Discussion

The purpose of this study was to construct the clusters of intimate relationship social support among trauma-exposed chronic pain patients. We identified three types. Type 1 indicated high overall support in all categories, type 2 indicated high emotional and informational support, while type 3 showed unstable support. Type 2 (who had high emotional and informational support) was associated with higher trauma exposure and PTSD. They also tended to be comprised of individuals who were non-familial (friends or other types of relationships) but there was no significant relationship between familial relations and intimate relationship clusters.

Studies show that individuals with chronic pain are more likely than other groups to rely on their most intimate relationships to play a caregiving role [23]. Caregivers and the individuals they care for do not engage in equal exchanges or level of contribution, and benefit is notequal [24]. The caregiver, according to social exchange theory, will thus be more likely to be dissatisfied with the relationship and will find that the relationship brings distress [25].

Chronic pain patients exposed to potentially traumatic events do benefit from high levels of social support [26]. They experience fewer symptoms of PTSD [27], reduced interpersonal conflict, reduced occupational stress, and a reduced likelihood of stress-related psychiatric disorders like depression and anxiety [28]. Nevertheless, the dependence of chronic pain patients on caregivers for instrumental and emotional support, along with social withdrawal, numbing, and excessive anger inherent to PTSD, can erode relationships over time [29,30]. These relationships themselves also might be source of traumatic exposure. The gradual decline of social support may leave individuals with chronic pain more vulnerable to additional potentially traumatic events and may further contribute to distress, depression, and anxiety. This dynamic, to some extent, may have been reflected among the two types with lower social support (types 2 and 3) in our sample. These two clusters might be operating in similar conditions. Type 2, given their level of trauma and PTSD, may have severed ties with familial relationships who may have provided support early on as exposure to potentially traumatic events occurred or who might have been the source of the exposure.

Clinicians should consider chronic pain patients’ sense of equity within intimate relationships. Part of treating chronic pain patients is related to restoring a sense of equity with their intimate relationships by involving their intimate relationships in determining ways in which the patient can reduce their benefits (rely less on caregiver), do more things for the caregiver or do things differently for the caregiver. When significant traumatic events and PTSD has increased the chronic pain patients’ level of irritability, anger, and willingness to engage in intimacy, then PTSD screening and additional cognitive therapies like cognitive processing therapy for couples with mental health professionals should be considered.

Several limitations were present in this study. First, the sample size is very low and is concentrated in a Midwestern city where the majority of residents were either White or African American. Because of limitations related to power, we were not able to use more sophisticated statistical methods to assess the role of potential confounding factors, such as income, health conditions, etc. Thus, results-related clusters of social support should be interpreted with caution. These results are preliminary and could change if additional subjects were added to the existing dataset. Nevertheless, this study is novel because it is one of the first to determine the qualities of chronic pain patients’ intimate relationships. Chronic pain patients need individuals to provide caregiving and tend to be more likely to have trauma exposure and PTSD, which puts additional stresses on these relationships. Therefore, an exploratory study of the most intimate social support networks is useful for understanding chronic pain patients needs and determining whether to integrate social support arrangements into treatment planning. Future studies utilizing larger samples from a more diverse geographic locations could build on these findings. Future studies could also use qualitative methodology to derive a better understanding of relationship problems of chronic pain patients with and without trauma exposure.

## 5. Conclusions

In this study, we constructed three clusters of intimate relationship social support among trauma-exposed chronic pain patients. We found that a significant proportion had very supportive intimate relationship social networks. However, the other two groups experienced varying degrees of stress in their relations plus less instrumental support. Clinicians should ask chronic pain patients with known trauma about the status of their social support networks in order to determine whether they are receiving appropriate support to enhance functioning, health behavior and future chronic pain outcomes.

## Figures and Tables

**Table 1:** Characteristics of the sample.

Characteristics	Statistic
Age, mean (SD)	52.9 (12.7)
Gender, # (%)	
Male	16 (39.0)
Female	25 (61.0)
Race/Ethnicity, # (%)	
White	21 (51.2)
African American	20 (48.8)
Other	0 (0.0)
Married or cohabiting, # (%)	17 (30.1)
Annual income, # (%)	
Less than $5000	5 (12.2)
$5000-$10,000	19 (46.3)
$20,000-$49,999	10 (24.4)
$50,000 and over	7 (17.1)
Education, # (%)	
Elementary/Junior High	3 (7.3)
High School Graduate, GED	9 (22.0)
Vocational Tech, Associates	19 (46.3)
Bachelor’s/Graduate School	10 (24.4)
Employed, # (%)	10 (18.2)
Past Trauma Exposure, mean (SD)	100% (range 1–19 traumatic events)
PTSD, # (%)	11 (26.8)
Depression, # (%)	10 (24.4)
Anxiety, # (%)	24 (58.5)
Pain, mean (SD)	5.1 (2.1)

**Table 2:** Clusters of social support in relationships of individuals with chronic pain.

Forms of Support	Cluster 1 (n = 17)	Cluster 2 (n = 9)	Cluster 3 (n = 15)
Instrumental Support	100%	30%	45%
Informational Support	100%	100%	13%
Affirmation Support	100%	100%	27%
Confidant Support	100%	78%	47%
Emotional Support	100%	89%	53%
Fun Support	100%	56%	40%

**Table 3:** Mean Level of Relationship stress by clusters of social support relationships.

Forms of Stressors (1 to 5)	Cluster 1 (n = 17)	Cluster 2 (n = 9)	Cluster 3 (n = 15)
Extent to which person makes your life more stressful	1.94	2.44	3.33
Extent to which person is critical and disapproving of you	1.88	1.67	3.00
Extent to which you don’t measure up to person’s expectations of you	1.94	2.0	2.60
Extent to which person puts too many demands on you	1.65	1.78	2.67
Extent to which person is verbally aggressive with you	1.47	1.67	2.27
Extent to which person is physically aggressive with you	1	1	1

**Table 4:** Descriptions of most intimate relationship by cluster group

Cluster	Interview Focus	Quote 1	Quote 2	Quote 3
1	Stress	She lacks patience, tendency for [mono-vision]. She’s a one-way/one-way only person, lack of empathy/compassion for others	We’re both Capricorns; she likes to try to finish my sentences; makes me feel like she’s invading my space—always wants to insert her opinion when it isn’t wanted/needed	She still commutes from Waterloo to Manchester on 270 every day. I’m afraid for her. Wish she would retire and get a new car. Still works 40 h a week
	Verbal aggression	I might have to fuss every once in a while, but that doesn’t happen real often	Her interpretation sometimes different than what I say; I tell her hate when she does that; 2–3 times/yr	I’m pretty sure he pushes my buttons to get me to react when drunk
	Positive Influence	Once in a great while she’ll say—let’s go for a walk, I did research w/nutritionist. I do all the cooking, I make/influence healthy lifestyle choices	Encourages me to quit smoking. Been trying to quit for a year. I tell him to keep trying/encouraging. “He’s a fantastic son.”	She’s always after me to eat better, more frequently
	Contributes to unhealthy choices	She wants me to buy her ice cream, take her to KFC, will buy candy; makes me the bad guy	If I was to go to his house and stay I may indulge in drinking more	Mostly he doesn’t care about lifestyle; just whatever for the moment
2	Stress	She has had multiple strokes; very stressful	Not always in the best physical health. She’s my play mother. I worry about her. I check on her	Unpredictable; kicked me out of the house; we argue a lot; don’t trust him; friends don't like me
	Verbal aggression	Very opinionated and outspoken	Calls me disgusting names—throw things	I’ll tend to yell at him if I get too angry
	Positive Influence	Insisting on my meds, to continue them, voicing concern about me being out in the cold about financial situation. Offers help	Very supportive in me coming out as transgender. Helps to see things w/less pessimism. “She is my Buddha”	Reminds me of medical appointments, pay bills, budgeting; take prescription
	Contributes to unhealthy choices	Not eating healthy foods	Encourage me to drink more than I should when he makes me angry	No exercise, no social life, no friends. I spend alot of time caring for him, waiting on him when he’s sick
3	Stress	Spouse resents me for being sick; talks down to me; threatens divorce...	Every time she has a problem I have to solve it. She depends on me financially	He’s just an ass. I don’t understand why I can’t help with things or do things
	Verbal aggression	He couldn’t take me telling him the truth; he sometimes feels I am critical.	Upset with him for not helping me when he’s created a difficult situation. I don’t have her car because of him	The nonsense. I don’t want to listen to his B.S., lies, and anything to do with his medical issues is all drug seeking
	Positive Influence	Let’s go on a diet, let’s go to the gym—always trying to get me physically active; wants to know what I’m eating; helped me quit smoking cigarettes; encourages stop doing other unhealthy things	Affirming, reminds me of successes and medications.	Discourages drinking when in a social drinking situation; asks if I took meds or got meds from doc
	Contributes to unhealthy choices	The other day after cancer surgeon appointment was so stressed needed sugar bought soda and donut	Both have bad habits and they reinforce each others bad habits (eating/exercise)	Tells me to do without. “Go walk to get it.” Doesn’t understand dangers of walking/falling for me. His girlfriend is very needy-why he didn’t help me after surgery
